# Colonization of methicillin-resistant *Staphylococcus* species in healthy and sick pets: prevalence and risk factors

**DOI:** 10.1186/s12917-023-03640-1

**Published:** 2023-07-18

**Authors:** Marta Miszczak, Agnieszka Korzeniowska-Kowal, Anna Wzorek, Andrzej Gamian, Krzysztof Rypuła, Karolina Bierowiec

**Affiliations:** 1 Department of Epizootiology and Clinic of Birds and Exotic Animals, Wrocław University of Environmental and Life Sciences, Wrocław, Poland; 2 Department of Immunology of Infectious Diseases, Ludwik Hirszfeld Institute of Immunology and Experimental Therapy, Wrocław, Poland

**Keywords:** *Staphylococcus*, Methicillin-resistant, Cats, Dogs, Risk factors

## Abstract

**Background:**

The characterization of staphylococcal species that colonize pets is important to maintain animal health and to minimize the risk of transmission to owners. Here, the prevalence of *Staphylococcus* spp*.* and methicillin resistance was investigated in canine and feline isolates, and risk factors of staphylococcal colonization were determined. Pets were examined and separated into four groups: (1) healthy dogs, (2) healthy cats, and (3) dogs and (4) cats with clinical signs of bacterial infections of skin, mucous membranes, or wounds. Specimens were collected by a veterinary physician from six anatomic sites (external ear canal, conjunctival sacs, nares, mouth, skin [groin], and anus). In total, 274 animals (cats *n* = 161, dogs *n* = 113) were enrolled.

**Results:**

*Staphylococcus* species were highly diverse (23 species; 3 coagulase-positive and 20 coagulase-negative species), with the highest variety in healthy cats (19 species). The most frequent feline isolates were *S. felis* and *S. epidermidis*, while *S. pseudintermedius* was the most prevalent isolate in dogs*.* Risk factors of staphylococcal colonization included the presence of other animals in the same household, medical treatment within the last year, and a medical profession of at least one owner. Methicillin resistance was higher in coagulase-negative (17.86%) compared to coagulase-positive (1.95%) staphylococci. The highest prevalence of methicillin-resistant CoNS colonization was observed in animals kept in homes as the most common (dogs and cats).

**Conclusions:**

The association of methicillin-resistant CoNS colonization with animals most often chosen as pets, represents a high risk of transmission between them and owners. The importance of nosocomial transmission of CoNS was also confirmed. This information could guide clinical decisions during the treatment of veterinary bacterial infections. In conclusion, the epidemiologic characteristics of CoNS and their pathogenicity in pets and humans require further research.

## Background

Staphylococci colonize both living organisms and inanimate surfaces, and can survive adverse environmental conditions. The *Staphylococcus* genus is comprised of two groups that are categorized by their production of the enzyme coagulase, constituting coagulase-positive (CoPS) and coagulase-negative staphylococci (CoNS) [1]. The greatest staphylococcal threat to human health is posed by the CoPS *S. aureus*. In dogs, and to a lesser extent in cats, a similar risk is associated with *S. pseudintermedius* [2, 3]. The most prevalent CoNS species that colonize humans are *S. epidermidis, S. haemolyticus, S. capitis, S. hominis,* and *S. simulans.* In comparison, the most common species in pets are *S. felis,* the *S. sciuri* group (including *S. lentus*), *S. capitis*, and *S. cohnii* [4, 5]. Until recently, CoNS were considered non-pathogenic or culture-based contaminants; however, this heterogeneous group is now considered clinically significant because of virulence factors and pathogenicity [1, 5–7].

Increasing staphylococcal virulence, especially among CoNS, is of concern. The transmission of virulence factors through horizontal gene transfer may confer drug resistance and biofilm production, which confound the treatment of staphylococcal infections [8–11]. Staphylococci can also survive intracellularly, and thus evade host immune defenses [4, 12, 13].

Both transient and chronic staphylococcal carriage have been described in multiple host species [14–16]. Staphylococcal colonization of healthy people and animals occurs at many cutaneous and mucosal sites including ears, conjunctival sacs, nares, mouth, skin, and anus [3, 4, 11, 17–20]. Staphylococcal infections of animals involve traumatic and surgical wounds; the bloodstream (especially in the presence of infected intravascular devices); the external ear canal; the upper respiratory, urinary, and genital tracts; bone and bone marrow, conjunctiva, and skin [1, 4, 21–26]. These infections may feature purulent exudate, tissue necrosis, and even sepsis [27–29]. Staphylococcal infections of humans include furunculosis, mastitis, conjunctivitis, keratitis, dacryocystitis, bacteremia, and superantigen-mediated diseases such as scalded skin and toxic shock syndromes [16, 20].

Risk factors of infections due to methicillin-resistant staphylococci (MRS) in dogs and cats include prior hospitalization of the animal, frequent visits to the veterinary office, administration of glucocorticosteroids, and antibiotic treatment [23, 30]. Also, equipment in veterinary clinics can be contaminated with staphylococci [29, 31–33].

This study evaluated the prevalence of *Staphylococcus* spp. in healthy and sick dogs and cats. The predilection of different anatomic sites to staphylococcal colonization was investigated, along with risk factors of colonization. Drug resistance was also explored, with a focus on methicillin resistance.

## Results

### Study population.

From 2019 to 2021, 274 animals were examined at the Department of Epizootiology and Clinic of Bird and Exotic Animals, Faculty of Veterinary Medicine, Wrocław University of Environmental and Life Sciences, Poland. Cats and dogs were assigned to four groups based on data obtained from clinical examinations and diagnostic interviews of owners. The groups were: healthy cats (*n* = 120), healthy dogs (*n* = 64), sick cats (*n* = 41), and sick dogs (*n* = 49). Sick animals were diagnosed with at least one of the following conditions: conjunctivitis, upper respiratory tract disease, and skin or wound infection.

### Isolation of *Staphylococcus* spp.

*Staphylococcus* spp. were isolated from 36.81% (265/720) of samples from healthy cats, 32.75% (94/287) of samples from sick cats, 36.46% (140/384) of samples from healthy dogs, and 34.69% (119/343) of samples from sick dogs. Detailed data on positive sampling are presented in Table 1.

**Table 1:** Positive sampling from collected material

Animal groups	No. of animals	No. of samples obtained from animals	No. of *Staphylococcus* isolates from samples	Positive sampling (Cl 95%)
Healthy Cats	120	720	265	36.81% (33.28–40.33)
Sick Cats	41	287	94	32.75% (27.32–38.18)
Healthy Dogs	64	384	140	36.46% (31.64–41.27)
Sick Dogs	49	343	119	34.69% (29.66–39.73)
Total	274	1734	618	35.64% (33.39–37.89)

*Cl* Confidence level

At least one *Staphylococcus* species was isolated from 81.75% (224/274) of animals. Twenty-three *Staphylococcus* species were isolated in total. The highest variety of *Staphylococcus* species was observed in healthy cats (19 species). The most frequently observed species in relation to the number of all staphylococcal isolates obtained from a given group of animals (both healthy and sick) were *S. felis* (n = 90; 25.07%; Cl 95%: 20.59–29.55%) and *S. epidermidis* (n = 65; 18.11%; Cl 95%: 14.12–22.09%) in cats, and *S. pseudintermedius* (*n* = 138; 53.28%; Cl 95%: 47.21–59.36%) and *S. epidermidis* (*n* = 42; 16.22%; Cl 95%: 11.73–20.71%) in dogs.

**Fig. 1:**
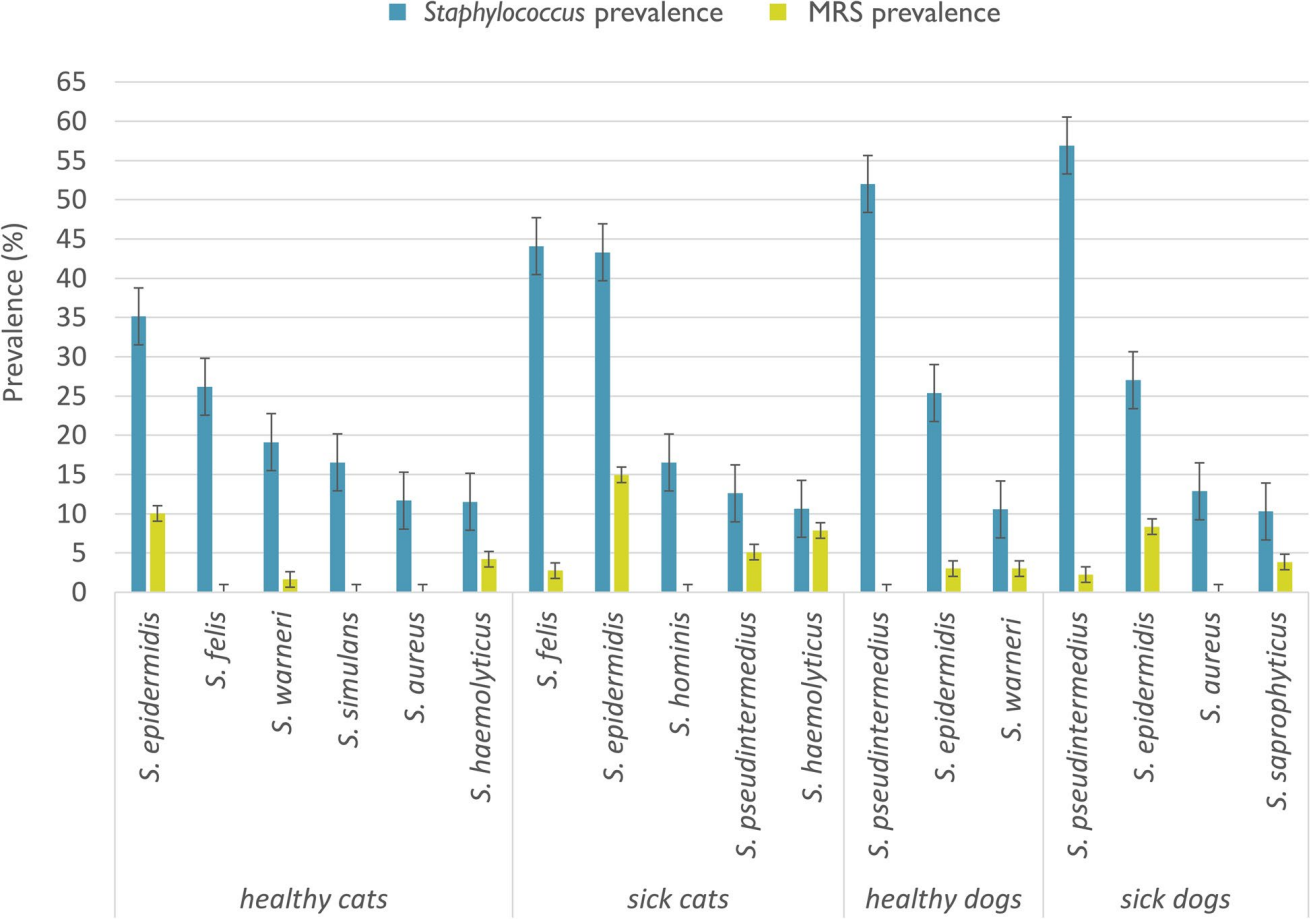
Prevalence of frequently isolated *Staphylococcus* spp. and methicillin-resistant isolates

The prevalence rates of the most frequently isolated staphylococci in each animal group are shown in Fig. 1. Only *S*. *felis* in cats and *S. pseudintermedius* in dogs were isolated from all swabbed anatomic sites. Data on the prevalence of staphylococcal species and their anatomic distributions are presented in Tables 2 and Table 3. The numbers of CoPS and CoNS strains obtained from each group are presented in Table 4.

**Table 2:** Prevalence of *Staphylococcus* species in healthy and sick cats

		Skin % (CI 95%)		Nares % (CI 95%)		Conjunctival sac % (CI 95%)		External auditory canal % (CI 95%)		Oral cavity % (CI 95%)		Anus % (CI 95%)		Wounds % (CI 95%)	
Species	n	HC	SC	HC	SC	HC	SC	HC	SC	HC	SC	HC	SC	HC	SC
*S. aureus*	26	0.81 (0.27–1.49)	0	5.70 (4.07–7.46)	2.36 (0.79–4.33)	2.59 (1.49–3.80)	0	0.81 (0.27–1.49)	2.76 (0.79–4.73)	6.78 (5.02–8.68)	3.54 (1.57–5.91)	0	0	0	2.76 (0.79–5.12)
*S. caprae*	1	0	0	0	0	0	0	0	0	0.95 (0.27–1.76)	0	0	0	0	0
*S. cohnii*	8	2.44 (1.36–3.66)	0	0.81 (0.27–1.49)	2.36 (0.79–4.33)	0.68 (0.14–1.36)	0	0.95 (0.27–1.76)	0	0	0	0	2.76 (0.79–4.72)	0	0
*S. condimenti*	1	0	0	0	0	0.95 (0.27–1.76)	0	0	0	0	0	0	0	0	0
*S. epidermidis*	65	8.41 (6.38–10.45)	9.45 (5.91–12.99)	8.55 (6.65–10.58)	2.76 (0.79–5.12)	15.33 (12.89–17.91)	11.02 (7.48–14.96)	8.68 (6.78–10.72)	22.83 (17.72–27.95)	5.83 (4.21–7.60)	5.12 (2.76–7.87)	4.07 (2.71–5.56)	2.36 (0.79–4.33)	0	0
*S. equorum*	1	0.81 (0.27–1.49)	0	0	0	0	0	0	0	0	0	0	0	0	0
*S. felis*	90	2.44 (1.36–3.66)	6.30 (3.54–9.45)	10.85 (8.68–13.16)	24.80 (19.69–30.31)	8.27 (6.38–10.31)	14.17 (10.24–18.50)	12.21 (9.91–14.65)	16.54 (12.20–21.26)	5.56 (3.93–7.33)	11.02 (7.48–14.96)	7.46 (5.70–9.50)	5.12 (2.76–7.87)	0	5.12 (2.76–7.87)
*S. haemolyticus*	19	1.63 (0.81–2.58)	2.76 (0.79–4.72)	2.44 (1.36–3.66)	0	2.44 (1.36–3.66)	5.12 (2.76–7.87)	3.26 (2.04–4.61)	2.76 (0.79–5.12)	1.76 (0.81–2.71)	0	0.81 (0.27–1.49)	0	0	0
*S. hominis*	21	3.39 (2.17–4.75)	5.91 (3.15–9.06)	1.63 (0.81–2.58)	2.76 (0.79–5.12)	2.44 (1.36–3.53)	5.12 (2.36–7.87)	1.63 (0.81–2.58)	2.36 (0.79–4.33)	0	9.06 (5.51–12.99)	0.81 (0.27–1.49)	0	0	0
*S. lentus*	2	0	2.76 (0.79–5.12)	0	0	0	0	0.95 (0.27–1.76)	0	0	0	0	0	0	0
*S. lugdunensis*	2	0	0	0	0	1.09 (0.41–1.90)	0	0	0	0	0	0.81 (0.27–1.49)	0	0	0
*S. pasteuri*	1	0	0	0.81 (0.27–1.49)	0	0	0	0	0	0	0	0	0	0	0
*S. pettenkoferi*	1	0	0	0	0	0.81 (0.27–1.49)	0	0	0	0	0	0	0	0	0
*S. pseudintermedius*	20	0.95 (0.27–1.76)	2.76 (0.79–5.12)	1.63 (0.81–2.58)	5.12 (2.76–7.87)	1.77 (0.95–2.71)	5.12 (2.76–7.87)	0	5.51 (2.76–8.28)	0.81 (0.27–1.49)	5.12 (2.76–7.87)	0	2.76 (0.79–4.72)	0	0
*S. saprophyticus*	12	1.76 (0.81–2.71)	0	0	5.12 (2.76–7.87)	0.95 (0.27–1.76)	0	2.44 (1.36–3.53)	0	0.81 (0.27–1.49)	0	0	0	0	0
*S. sciuri*	1	0	0	0	2.36 (0.79–4.33)	0	0	0	0	0	0	0	0	0	0
*S. simulans*	31	2.44 (1.36–3.66)	2.36 (0.79–4.33)	3.26 (2.04–4.61)	6.30 (3.54–9.45)	0.81 (0.27–1.49)	2.76 (0.79–5.12)	10.04 (7.87–12.35)	0	2.44 (1.36–3.66)	0	1.63 (0.81–2.58)	2.76 (0.79–5.12)	0	2.76 (0.79–4.72)
*S. succinus*	1	0.81 (0.27–1.49)	0	0	0	0	0	0	0	0	0	0	0	0	0
*S. warneri*	40	6.78 (5.02–8.68)	7.48 (4.33–11.02)	3.26 (2.04–4.61)	2.36 (0.79–4.33)	9.91 (7.87–12.08)	0	2.58 (1.49–3.80)	2.36 (0.79–4.33)	3.93 (2.58–5.43)	2.36 (0.79–4.33)	0	0	0	0
*S. xylosus*	16	0	0	4.07 (2.71–5.56)	2.76 (0.79–4.73)	1.76 (0.95–2.71)	2.76 (0.79–4.72)	1.63 (0.81–2.58)	0	3.26 (2.04–4.61)	0	0	0	0	0

*n* number of isolated strains, *HC* Healthy cats, *SC* Sick cats (Sick animals were diagnosed with at least one of the following conditions: conjunctivitis, upper respiratory tract disease, and skin or wound infection), *CI 95%* confidential interval 95%

**Table 3:** Prevalence of *Staphylococcus* species in healthy and sick dogs

		Skin % (CI 95%)		Nares % (CI 95%)		Conjunctival sac % (CI 95%)		External auditory canal % (CI 95%)		Oral cavity % (CI 95%)		Anus % (CI 95%)		Wounds % (CI 95%)	
Species	n	HD	SD	HD	SD	HD	SD	HD	SD	HD	SD	HD	SD	HD	SD
*S. aureus*	19	0	4.18 (2.25–6.43)	4.52 (2.51–6.78)	8.68 (5.79–11.90)	1.51 (0.50–2.76)	2.25 (0.64–4.18)	1.76 (0.50–3.27)	0	1.51 (0.50–2.76)	6.43 (3.86–9.32)	1.76 (0.50–3.27)	0	0	4.50 (2.25–6.75)
*S. capitis*	4	1.51 (0.50–2.76)	1.93 (0.64–3.54)	1.51 (0.50–2.76)	0	0	0	1.51 (0.50–2.76)	0	0	0	0	0	0	0
*S. cohnii*	3	0	0	0	0	0	0	3.02 (1.51–4.77)	0	0	0	0	0	0	2.25 (0.64–3.87)
*S. delphini*	2	0	0	0	0	0	0	1.51 (0.50–2.76)	0	0	0	0	0	0	2.25 (0.64–3.87)
*S. epidermidis*	42	10.55 (7.79–13.57)	8.04 (5.14–11.25)	4.52 (2.51–6.53)	6.11 (3.54–9.00)	8.79 (6.03–11.56)	10.61 (7.40–14.15)	4.52 (2.51–6.53)	6.11 (3.54–9.00)	4.52 (2.51–6.53)	1.93 (0.64–3.54)	0	0	0	4.18 (2.25–6.43)
*S. felis*	1	0	0	0	0	0	0	0	1.93 (0.64–3.54)	0	0	0	0	0	0
*S. haemolyticus*	9	0	0	1.51 (0.50–2.76)	0	3.77 (2.01–5.78)	0	3.02 (1.51–4.77)	0	1.51 (0.50–2.76)	0	3.02 (1.51–4.77)	0	0	0
*S. hominis*	3	1.51 (0.50–2.76)	0	0	0	0	0	0	1.93 (0.64–3.54)	0	0	1.76 (0.50–3.27)	0	0	0
*S. lentus*	1	1.51 (0.50–2.76)	0	0	0	0	0	0	0	0	0	0	0	0	0
*S. lugdunensis*	3	0	0	1.51 (0.50–2.76)	0	1.51 (0.50–2.76)	0	0	0	1.51 (0.50–2.76)	0	0	0	0	0
*S. pseudintermedius*	138	13.82 (10.55–17.34)	14.79 (10.93–18.97)	16.83 (13.32–20.60)	28.94 (24.12–34.08)	12.06 (9.05–15.33)	29.90 (24.76–35.05)	12.56 (9.30–15.83)	17.36 (13.18–21.54)	26.38 (22.11–30.90)	22.83 (18.33–27.33)	27.89 (23.62–32.41)	17.36 (13.18–21.54)	0	9.00 (6.10–12.22)
*S. saprophyticus*	8	0	0	1.51 (0.50–2.76)	1.93 (0.64–3.54)	0	6.11 (3.54–9.00)	1.51 (0.50–2.76)	1.93 (0.64–3.54)	0	0	0	0	0	2.25 (0.64–3.86)
*S. schleiferi ssp schleiferi*	3	0	0	0	0	0	1.93 (0.64–3.54)	0	1.93 (0.64–3.54)	0	0	0	0	0	1.76 (0.50–3.02)
*S. simulans*	10	1.51 (0.50–2.76)	2.25 (0.64–4.18)	3.02 (1.51–4.77)	1.93 (0.64–3.54)	1.51 (0.50–2.76)	0	1.51 (0.50–2.76)	1.93 (0.64–3.54)	0	1.93 (0.64–3.54)	1.51 (0.50–2.76)	0	0	0
*S. warneri*	9	4.52 (2.51–6.78)	2.25 (0.64–3.86)	0	2.25 (0.64–4.18)	6.03 (3.77–8.54)	0	0	0	0	0	0	0	0	0
*S. xylosus*	4	1.51 (0.50–2.76)	0	3.02 (1.51–4.77)	0	0	0	0	0	0	0	0	0	0	1.93 (0.64–3.54)

*n* number of isolated strains, *HD* Healthy dogs, *SD* Sick dogs (sick animals were diagnosed with at least one of the following conditions: conjunctivitis, upper respiratory tract disease, and skin or wound infection), *CI 95%* Confidence interval 95%

**Table 4:** Number of CoPS and CoNS strains obtained from each group

	**Cats**		**Dogs**		Total
Isolates	Healthy	Sick	Healthy	Sick	
CoPS	28	18	80	79	205
CoNS	237	76	60	40	413
Total	265	94	140	119	**618**

### Detection of methicillin resistance

A total of 597 staphylococcal isolates (96.6% of total collected (597/618)) that had grown after storage at -80 °C were tested for methicillin resistance. From a group of CoNS isolates (from both healthy and sick cats and dogs) (*n* = 392), 70 isolates were oxacillin-resistant (17.86%; Cl 95%: 14.07–21.65%). In a group of CoPS isolates from both healthy and sick animals (*n* = 205), only four isolates exhibited oxacillin resistance (1.95%; Cl 95%: 0.06–3.84%). Overall, 22.36% (Cl 95%: 15.92–28.80%) of both healthy and sick cats and 16.81% (Cl 95%: 9.92–23.71%) of dogs were colonized with MRS. The prevalence rates of the most frequently isolated MRS are shown in Fig. 1.

### Statistical analysis

A questionnaire regarding risk factors was completed and returned by the owners of 80% of animals. Analysis of these questionnaires identified the presence of other animals in the same household, medical treatment within the last year, and a medical profession of at least one owner as important risk factors of staphylococcal colonization. CoPS colonization was unrelated to medical professions of owners. Breed, age, and sex were unrelated to the isolation of *Staphylococcus* spp. Animals that lived with other pets in the same household carried more *Staphylococcus* species (17 in cats, 16 in dogs) compared to those kept individually (11 in cats, 12 in dogs). On the contrary, *S. warneri* and *S. felis* were isolated more frequently from individually-kept cats and dogs, respectively (Table 5).

**Table 5:** Statistical analysis of risk factors associated with staphylococcal colonization

Variable		Test	Animals	Species	*P* value	OR	95%CI
Presence of children under 12 years of age in the household		Wilcoxon	Cats	*S. xylosus*	0.01	-	-
			Dogs	*S. epidermidis*	0.04		
				*S. aureus*	0.02		
Family member works in healthcare		Fisher	Cats	*S. epidermidis*	0.04	0.27	0.05–1
				*S. warneri*	0.04	0	0–0.99
			Dogs	*S. simulans*	0.01	13.12	1.69–158.59
Family member works in veterinary healthcare		Fisher	Dogs	*S. simulans*	0	0	0–1.77
Hospitalization of an owner in the previous year		Fisher	Dogs	*S. simulans*	0.01	13.52	1.42–662.82
for more than 7 days			Cats	*S. pseudintermedius*	0.02	8.26	1.13–48.63
Treatment of pet under investigation in the previous year		Chi-squared*	Cats	*S. epidermidis*	0.04	2.35	1.01–5.54
		Fisher		*S. cohnii*	0.04	5.039	0.81–36.27
		Chi-squared*		*S. hominis*	0.04	3.53	1–12.26
		Fisher	Dogs	*S. saprophyticus*	0.05	0	0.87
Treatment of other pets in the previous year		Chi-squared*	Cats	*S. felis*	0	4.36	1.7–11.58
		Fisher	Dogs	*S. haemolyticus*	0.01	13.05	1.38–639.26
Number of other animals kept in the same household	In general	Wilcoxon	Cats	*S. warneri*	0.02	-	-
			Dogs	*S. simulans*	0		
	Dogs		Dogs	*S. haemolyticus*	0.03		
				*S. simulans*	0.01		
	Cats		Cats	*S. warneri*	0.01		
	Others		Cats	*S. felis*	0.02		
			Dogs	*S. simulans*	0		
Contact with other animals		Chi-squared*	Cats	*S. warneri*	0.01	0.2	0.05–0.78
		Fisher	Dogs	*S. pseudintermedius*	0.01	0.1	0–0.76
Cats with outdoor access		Chi-squared*	Cats	*S. xylosus*	0.01	5.12	1.37–21.33

*P value* Probability value, *Chi-squared∗* Degrees of freedom is 1, *OR* Odd ratio, *CI* Confidential interval

## Discussion

This study demonstrated that both healthy and sick pets carry both CoPS and CoNS; however, some species were more highly associated with selected groups of animals. The prevalence of *Staphylococcus* spp. was nearly 82%, supporting previous studies [34]. Healthy cats exhibited the highest variety of staphylococcal isolates. This result contradicted those obtained by Ma et al. [35], in which dogs carried the most diverse range of *Staphylococcus* spp. This discrepancy may have been due to the disproportionately large sample size of healthy cats in our study.

Our finding that animals sharing the same households carried more *Staphylococcus* species suggests transmission between animals living in groups. The higher prevalence of *S. warneri* and *S. felis* in individually-kept cats and dogs, respectively, should be examined in further studies, because significantly fewer dogs were tested than cats. This is particularly interesting because *S. felis* is isolated primarily from cats [4, 5]. Unfortunately, we do not have information regarding previous cat contact of either the *S. felis*-positive dogs or their owners, and therefore the origin of their strains is unknown. Perhaps *S. warneri* and *S. felis* are at a competitive disadvantage against the predominant *Staphylococcus* species that colonize animals kept in groups, and are therefore more often observed in individually-kept animals.

Interestingly, CoPS were isolated more frequently from dogs (61.4%) compared to cats (12.8%), whereas more CoNS were isolated from cats (87.2%) compared to dogs (38.6%). Our results were similar to those of Abdel-Moein et al. [7] but contrasted with those of Sukur et al. [36]. The high rate of CoPS in dogs is associated with frequent carriage of *S. pseudintermedius*, as confirmed by our study and previous reports, while in cats both *S. aureus* and *S. pseudintermedius* are much less common [2, 3, 26]. This result is important when considering the high methicillin resistance rate of CoNS isolates worldwide [5, 7, 34]. Few studies have compared the virulence factors of CoNS and CoPS [1]; however, both groups are now considered major pathogens, due to drug resistance, possible horizontal gene transfer, and zoonotic potential [1, 4, 5, 7]. The highest methicillin resistance rate was observed among CoNS (nearly 18%) in the current study, in contrast to only 2% in the CoPS group. This result suggests that the pathogenicity of CoNS may be underrated in clinical practice. Becker et al. [4] described CoNS (from both humans and animals) as a reservoir of mobile genetic elements encoding β-lactam- and multidrug resistance. Abdel-Moein et al. [36] suggested that CoNS is a reservoir of resistance genes that can be transmitted to *S. aureus*, while Argemi et al. [37] showed that CoNS harbored multiple genes responsible for adhesion, biofilm formation, enzyme production, and the encoding of superantigens. These data are of concern, especially in the context of nosocomial infections [4, 5, 36, 37]. Our study showed that the medical profession (both veterinary and human medicine) of the owner, treatment received by the animal over the preceding year, and hospitalization of the owner had a more statistically significant influence on CoNS isolation (*S. cohnii, S. epidermidis, S. haemolyticus, S. hominis, S. simulans, S. warneri*) compared to CoPS. This shows that exposures to medical facilities, whether due to the owner’s occupation or the patient status of either owner or pet, increases the risk of CoNS colonization. Therefore, medical facilities should be considered as potential reservoirs of these bacteria. Interestingly, such associations were previously ascribed more often to CoPS. *Staphylococcus pseudintermedius* was isolated more frequently from cats that were treated over the preceding year. In contrast, isolation of *S. aureus* from the tested animals was not correlated to any factor, contradicting the assumption that it is a most highly pathogenic *Staphylococcus* species associated with nosocomial infections [38–40]. A growing body of evidence indicates that hospitals and veterinary offices are sources of highly pathogenic bacteria, such as CoPS (especially methicillin-resistant *S. aureus*) [8, 31, 32, 41–43]. This study demonstrates that, in fact, not only CoPS (as evidenced by the literature) but also CoNS may be transmitted from medical workplaces to households, and between people and animals. An analysis of colonization of human and pet animal pairs could be an interesting topic for further research.

Interestingly, the presence of children under 12 years of age in the household was a significant risk factor for *S. aureus* colonization of dogs. This phenomenon might be attributed to children maintaining close physical contact with dogs and having lower hygiene practices. *S. aureus* colonizing the skin and mucous membranes of children is presumably transmitted to dogs during close physical contact. This confirms that close contact between animals and their owners is a major risk factor for staphylococcal colonization, with both anthroponotic and zoonotic transmission being possible [42, 44–47].

*S. pseudintermedius* is isolated frequently from dogs globally [17, 28, 47]. Of note, in the current study, *S. pseudintermedius* was isolated more frequently from dogs that were in contact with other animals. Unexpectedly, outdoor cats were more likely to be colonized with *S. xylosus* compared to indoor-only cats; thus, the microbiota of these groups might differ, supporting the findings of Older et al. [48]. This finding might be explained by increased opportunities of outdoor cats for contact with other animals, people, and the external environment.

Despite investigating a large number of samples the current study did have some limitations. First, due to the disproportionately large sample size of healthy cats, it is difficult to accurately compare the prevalence of staphylococci species in other groups of animals. The low values may be due to the relatively small number of animals tested. Moreover, due to the very large total number of obtained *Staphylococcus* spp. strains, resistance testing at the genotypic level (by PCR) has not been performed. According to the Clinical and Laboratory Standards Institute (CLSI) Performance Standards for Antimicrobial Disk and Dilution Susceptibility Tests for Bacteria Isolated From Animals, supplement VET01S, for other *Staphylococcus* strains (excluding *S. aureus, S. lugdunensis, S. epidermidis, S. pseudintermedius* and *S. schleiferi*) oxacillin MIC breakpoints may overcall resistance, and some isolates for which the oxacillin MICs are 0.5–2 μg/mL may be *mecA* negative. These isolates could be tested for presence of *mecA* gene or for PBP2a, and if found negative, should be reported as methicillin (oxacillin) susceptible [49]. In this study, these additional methods were not performed. Finally, only oxacillin was used in the MIC resistance test. Considering the above, further research projects should be extended with these methods to obtain more complete results.

## Conclusions

This study confirmed that staphylococcal colonization is common in domesticated dogs and cats, with the most prevalent species being *S. pseudintermedius* in dogs and *S. felis* and *S. epidermidis* in cats. CoPS and CoNS were more often isolated from dogs and cats, respectively. Of importance, methicillin resistance was more prevalent in CoNS than in CoPS. The highest rate of methicillin resistance in CoNS isolates was observed in pets kept in homes, suggesting a high risk of both anthroponotic and zoonotic transmission. Consequently, physicians and veterinarians should educate their patients and pet owners on maintaining appropriate hygiene during contact with animals. The importance of nosocomial transmission of CoNS was also confirmed. This information could guide clinical decisions when veterinary patients are treated for bacterial infections. Particular attention should be paid to the role of CoNS in infections of the skin and mucous membranes and to the conduction of a thorough diagnostic evaluation (including bacteriological culture and antibiotic susceptibility testing) and rational drug selection. Unfortunately, CoNS are still considered by some clinicians as non-pathogenic commensals; therefore, their isolation from clinical specimens may not always lead to further analysis, antibiotic susceptibility testing, or targeted antibiotic therapy; resulting in adverse clinical outcomes and the spread of resistance and virulence factors. In conclusion, the epidemiologic characteristics of CoNS and their pathogenicity in pets and humans require further research.

## Materials and methods

### Study population and sampling procedures

This study extended the animal groups and number of collection sites used in a previous study [50], to obtain more details on staphylococcal colonization of pets. Pets were separated into four groups: (1) healthy dogs, (2) healthy cats, and (3) dogs and (4) cats with clinical signs of bacterial infections on the skin, mucous membranes, or wounds. Animals were only included after receiving permission from the owners to collect samples. Each owner was asked to complete a survey about the pet and household (home environment).

The research project was submitted to the Local Ethics Committee for Animal Experiments in Wrocław, Hirszfeld Institute of Immunology and Experimental Therapy, Polish Academy of Sciences, Poland. Due to the project’s proposed noninvasive sampling methods, the Ethics Committee qualified the study as research, thus exempting it from any further approval from the Ethics Committee. All methods described were approved by Wroclaw University of Environmental and Life Sciences, and were performed in compliance with the relevant guidelines and regulations for good laboratory practices. Each pet owner submitted informed consent to participate in this study, and completed the proper documentation.

Specimens were collected from six anatomic sites of each pet by a veterinary physician at study admission (immediately after obtaining consent of the animal owner for its study participation); these included the external ear canal, conjunctival sacs, nares, oral cavity, skin (groin), and anus. In the groups of sick animals, an extra swab was collected from the diseased wound or skin, if present.

### Isolation and identification of *Staphylococcus* spp. from samples

The collected material was placed in 2 ml of liquid brain–heart infusion broth (BHI) (Oxoid, Basingstoke, United Kingdom). The samples were then incubated at 37 °C for 24 h and then submitted for species identification. *Staphylococcus* spp. were isolated and identified from samples following an existing method [50]. One microliter of bacterial BHI stock was sub-cultured in mannitol-salt agar and Columbia blood agar plate (Oxoid, Basingstoke, United Kingdom). The plates were then incubated for 24 h at 37 °C. If the culture result was uncertain (only single visible colonies) or negative (no bacterial growth on the plate), incubation was extended to 48 h. After this time, the absence of bacterial growth was considered a negative culture result. Preliminary identification of staphylococci was based on colony morphology on Columbia blood agar and mannitol salt agar simultaneously. Bacterial colonies which grew on Columbia blood agar, diameter 2–6 mm, round, smooth, glistening, opaque, white or more or less yellow pigmented (grey-yellow, yellow-orange, gold), with or without beta-hemolysis zones were selected for subsequent identification. On the mannitol-salt agar plates, yellow colonies with surrounding yellow medium were suspected as CoPS and red colonies without medium color change were suspected as CoNS. The colonies used for the identification were obtained after comparing the growth of bacteria simultaneously on both the used media in order to minimize the risk of making a mistake in the diagnosis. The preliminary identification of suspected strains included Gram staining and detection of enzyme production (coagulase tube test; IBSS Biomed, Cracow, Poland). In case of morphologically distinguishable staphylococcal colonies were visible (maximum 5 colonies), these were cultured separately again in solid medium (Columbia blood agar) to obtain pure colonies.

A single colony from selected, and pure strains were further identified by matrix-assisted laser desorption ionization time of flight mass spectrometry (MALDI-TOF MS), following the method of Król et al. [51]. Raw spectra were processed using MALDI Biotyper OC v.3.1. software (Bruker Daltonik GmbH, Bremen, Germany). The results were classified to the species level using score values proposed by the manufacturer. The following identification scores were used: < 1.7 = no reliable identification; 1.7–1.999 = probable identification to the genus level; 2.0–2.299 = secure genus identification, probable species identification; and 2.3–3.0 = highly probable species identification. Scores ≥ 2.0 were considered as acceptable species-level identification [51]. All species were assigned after obtaining a score higher than 2.0. The indicated strains were stored for further analysis in 1 ml of bacterial stock in BHI with 15% glycerol at -80 °C for up to 12 months.

### Detection of methicillin resistance

All staphylococcal isolates that had grown after storage in -80 °C were tested for methicillin resistance. Methicillin resistance was detected using oxacillin with the broth microdilution method, using polystyrene, sterile titer plates (FL MEDICAL, Torreglia (PD), Italy). The examined strains were inoculated in cation-adjusted Mueller–Hinton broth (Oxoid, Basingstoke, United Kingdom) and in a dilution series of 4, 2, 1, 0.5, 0.25, and 0.125 µg/mL oxacillin stock solutions (TOKU-E, Gent, Belgium) in water [49, 52]. Inoculum (colony suspension, equivalent to a 0.5 McFarland Standard) was prepared according to the standard broth microdilution procedure described in Clinical and Laboratory Standards Institute (CLSI) Performance Standards for Antimicrobial Disk and Dilution Susceptibility Tests for Bacteria Isolated From Animals, supplement VET01S [49]. Samples were incubated for 24 h at 37 °C. Methicillin-resistant strains were detected using CLSI criteria for each *Staphylococcus* species [49]. *S. aureus* ATCC 29213 was used as a negative control, and *S. aureus* ATCC 43300 (*mec*A-positive) was used as a positive control.

### Statistical analysis

Statistical analysis of potential risk factors of colonization by specific *Staphylococcus* spp. was based on information provided by owners. Animals were excluded if owners did not return or complete the questionnaire. Data on the characteristics of pets, along with their medical history and environmental living conditions, were compared with scores on the frequency of *Staphylococcus* spp. isolation and oxacillin resistance. Statistical analysis was conducted using the R statistical package (v 3.6.3.). The prevalence and confidence intervals of staphylococci and MRS were calculated using the bootstrap method. All data were analyzed using the Shapiro–Wilk test, Wilcoxon test, Kruskal–Wallis test, Chi-square tests, and Fisher's test. *P* < 0.05 was considered statistically significant.

## Data Availability

All data generated or analysed during this study are included in this published article.

## Abbreviations

CoPS: Coagulase-positive staphylococci
CoNS: Coagulase-negative staphylococci
BHI: Brain–heart infusion broth
MALDI-TOF MS: Matrix-assisted laser desorption ionization-time of flight mass spectrometry
CLSI: Clinical and Laboratory Standards Institute
MRS: Methicillin-resistant staphylococci
